# Antiretroviral postnatal prophylaxis to prevent HIV vertical transmission: present and future strategies

**DOI:** 10.1002/jia2.26032

**Published:** 2023-02-20

**Authors:** Martina Penazzato, Ivy Kasirye, Theodore Ruel, Irene Mukui, Adrie Bekker, Mohendran Archary, Philippa Musoke, Shaffiq Essajee, George K. Siberry, Mary Mahy, Daniele Simnoue, Beatriz Simione, Jennifer M. Zech, Angela Mushavi, Elaine J. Abrams, PNP meeting participants

**Affiliations:** ^1^ HIV Department World Health Organization Geneva Switzerland; ^2^ Department of Pediatrics University of California San Francisco San Francisco California USA; ^3^ Drugs for Neglected Diseases Initiative Nairobi Kenya; ^4^ Family Centre for Research with Ubuntu, Department of Paediatrics and Child Health Stellenbosch University Cape Town South Africa; ^5^ Department of Paediatrics University of KwaZulu‐Natal Durban South Africa; ^6^ Department of Paediatrics and Child Health Makerere University and MUJHU Research Collaboration Kampala Uganda; ^7^ United Nations International Children's Emergency Fund New York City New York USA; ^8^ Office of HIV/AIDS, Bureau of Global Health United States Agency for International Development (USAID) Washington DC USA; ^9^ Strategic Information Department UNAIDS Geneva Switzerland; ^10^ World Health Organization Cameroon Yaoundé Cameroon; ^11^ Ministry of Health Maputo Mozambique; ^12^ ICAP at Columbia University, Mailman School of Public Health and Department of Pediatrics, Vagelos College of Physicians & Surgeons Columbia University New York City United States; ^13^ Ministry of Health Harare Zimbabwe

**Keywords:** postnatal prophylaxis, paediatrics, HIV, antiretrovirals, drug formulations, vertical transmission

## Abstract

**Introduction:**

Maternal antiretroviral therapy (ART) with viral suppression prior to conception, during pregnancy and throughout the breastfeeding period accompanied by infant postnatal prophylaxis (PNP) forms the foundation of current approaches to preventing vertical HIV transmission. Unfortunately, infants continue to acquire HIV infections, with half of these infections occurring during breastfeeding. A consultative meeting of stakeholders was held to review the current state of PNP globally, including the implementation of WHO PNP guidelines in different settings and identifying the key factors affecting PNP uptake and impact, with an aim to optimize future innovative strategies.

**Discussion:**

WHO PNP guidelines have been widely implemented with adaptations to the programme context. Some programmes with low rates of antenatal care attendance, maternal HIV testing, maternal ART coverage and viral load testing capacity have opted against risk‐stratification and provide an enhanced PNP regimen for all infants exposed to HIV, while other programmes provide infant daily nevirapine antiretroviral (ARV) prophylaxis for an extended duration to cover transmission risk throughout the breastfeeding period. A simplified risk stratification approach may be more relevant for high‐performing vertical transmission prevention programmes, while a simplified non‐risk stratified approach may be more appropriate for sub‐optimally performing programmes given implementation challenges. In settings with concentrated epidemics, where the epidemic is often driven by key populations, infants who are found to be exposed to HIV should be considered at high risk for HIV acquisition. All settings could benefit from newer technologies that promote retention during pregnancy and throughout the breastfeeding period. There are several challenges in enhanced and extended PNP implementation, including ARV stockouts, lack of appropriate formulations, lack of guidance on alternative ARV options for prophylaxis, poor adherence, poor documentation, inconsistent infant feeding practices and in inadequate retention throughout the duration of breastfeeding.

**Conclusions:**

Tailoring PNP strategies to a programmatic context may improve access, adherence, retention and HIV‐free outcomes of infants exposed to HIV. Newer ARV options and technologies that enable simplification of regimens, non‐toxic potent agents and convenient administration, including longer‐acting formulations, should be prioritized to optimize the effect of PNP in the prevention of vertical HIV transmission.

## 1 INTRODUCTION

Despite decades of progress, vertical HIV transmission continues to occur. Globally, 85% of pregnant women living with HIV were receiving antiretroviral therapy (ART) and 150,000 new vertical HIV infections occurred in 2020, highlighting the failure to meet the 2020 global targets [1]. HIV transmission during pregnancy and the breastfeeding period can be linked to mothers with incident maternal HIV infections and established HIV infection who do not receive or continue ART [1].

Prevention and detection of new HIV infections in women during pregnancy and breastfeeding and retention of women living with HIV on ART remain challenging. Evidence indicates high HIV incidence during pregnancy and breastfeeding in Sub‐Saharan Africa (SSA) [2, 3]. Furthermore, retention in HIV care among pregnant and postpartum women with established HIV infection in low‐ and middle‐income countries is estimated to be 79.4% at 6 months after ART initiation and 74.5% at 12 months after initiation [4]. Globally, the average maternal postpartum disengagement from HIV care is 1.2% per month during the first 12 months and 0.7% per month from the 13th month to the end of breastfeeding [5]. Historically, the postpartum viral rebound has been observed in approximately one‐third of women starting efavirenz‐based ART during pregnancy who had initial viral suppression, and 22% of women receiving pre‐conception ART had detectable viremia at first antenatal visit [6, 7]. Whether the rates of sustained viral suppression in pregnant and postpartum women improve with the introduction of dolutegratvir‐based regimens is yet to be determined. Overall attrition (lost‐to‐follow‐up + death) of infants exposed to HIV from infant care programs is estimated at 25% by 2 months of age and 39% by 18 months of age with lost‐to‐follow‐up (LTFU) accounting for 17% of attrition by 2 months of age and 26% by 18 months of age. The majority of LTFU and overall attrition is estimated to occur within the first 6 months of follow‐up [8].

The combination of maternal ART with viral suppression throughout the period of transmission risk and infant antiretroviral (ARV) prophylaxis from birth through the first months of life is safe and effective, reducing HIV vertical transmission to very low rates [9, 10, 11, 12]. Based on limited evidence, a systematic review suggests that using 6–12 weeks of combination ARV regimens (“enhanced” postnatal prophylaxis [PNP]) in high‐risk infants reduces intrapartum transmission and that using “extended” prophylaxis (beyond 12 weeks) in breastfed infants reduces breastfeeding transmission rates when mothers are not on ART [12, 13]. However, whether there is an additional benefit of enhanced or extended (PNP) regimens in the context of effective maternal ART remains unclear [14].

To reduce the risk of HIV transmission in the postpartum period and optimize infant survival, current WHO guidelines recommend that mothers living with HIV are diagnosed early, receive effective treatment and exclusively breastfeed their infants for 6 months and then breastfeed with complementary foods for up to 2 years. In 2016, the WHO introduced risk stratification for HIV acquisition and recommendations for enhanced PNP for infants at increased risk of HIV acquisition. However, challenges in identifying high‐risk infants and administering PNP persist [15].

The WHO and the IMPAACT network hosted a workshop entitled “*Postnatal prophylaxis: optimizing research and accelerating access to innovation*” that aimed to accelerate the investigation of new strategies to prevent vertical transmission in the postnatal period. This workshop, held virtually through a series of meetings from May to December 2021, brought together academic researchers, clinical experts, women living with HIV, regulators, industry representatives, HIV programme managers, funders and other key stakeholders involved in the prevention of vertical transmission of HIV research and service delivery. The overall goal was to gain consensus on the approach to investigate innovative strategies for PNP and establish the next steps for the implementation of such studies. The first consultation was focused on reviewing the status of the implementation of PNP in multiple countries, the evolving landscape of maternal treatment in the same settings and developing key considerations for the design of effective PNP strategies tailored to the epidemic context.

This paper builds on the outcome of the first consultation in the series which examined current challenges and opportunities for the implementation of PNP in order to inform the design of future innovative strategies to deliver effective PNP to infants exposed to HIV.

## 2 DISCUSSION

### 2.1 Status of PNP implementation

WHO conducted a desk review of existing country PNP policies in 18^1^ of the 21 Start Free, Stay Free, AIDS Free priority countries [16]. All countries included enhanced PNP in their guidelines—61% for high risk and 39% for all infants. There was heterogeneity of enhanced PNP strategies with nine distinct combinations of ARV regimens and durations in use. Regimens included triple, dual and single ARV regimens. The duration of prophylaxis ranged from 4 weeks after birth to 6 weeks after cessation of breastfeeding. Seventeen countries provide enhanced PNP for at least 12 weeks after birth, including dual or triple ARVs for the initial 6 weeks followed by nevirapine alone for the remainder of the enhanced PNP period or dual or triple ARVs regimen for 12 weeks. Only 31% of the countries whose PNP policies were reviewed reported having any system in place to monitor the uptake or toxicity of prolonged/extended PNP [17].

Reported challenges in enhanced PNP implementation include ARV stockouts, lack of age‐appropriate formulations, lack of guidance on alternative ARV regimens, poor adherence and retention, low uptake of antenatal, maternity, postnatal and prevention of vertical transmission services, high turnover of trained health workers as well as limited coverage of maternal viral load testing and long turnaround time for results. Countries indicated challenges with PNP, including the lack of global guidance for PNP in infants whose breastfeeding mothers had episodes of poor adherence or persistent viremia as well as for situations where mothers receiving second‐ or third‐line ART regimens are likely to harbour virus resistant to ARVs used for PNP [17].

### 2.2 Voices of women living with HIV

Mothers‐2‐mothers (m2m) interviewed 20 women living with HIV among those accessing peer‐mothers services in three high burden countries (Uganda, Malawi and South Africa) with targeted questions to evaluate acceptability, preferences and challenges attached to the provision of PNP. Findings were synthesized, themes extracted and key messages shared with meeting participants in plenary. Women interviewed reported that mothers found it difficult to give nevirapine syrup for 6 weeks and even more difficult to give it for 12 weeks and that many did not return for refills after the first bottle. “Can they give us all syrups at birth so we do not have to come back many times?”: many mothers have not disclosed their HIV status because of fear of stigma and keeping infants on multiple syrups for 6–12 weeks with repeated facility visits may result in inadvertent undesired disclosure. Multiple visits were also challenging for working mothers. Mothers also reported that triple therapy beyond 6 weeks made them feel as if their babies already had HIV infection. Stockouts of ARVs were a concern and inconvenient for parents. Finally, COVID‐19 restrictions worsened the situation with increased transportation costs.

### 2.3 Is PNP still needed?

The ART landscape is rapidly evolving with approximately 60% of women entering pregnancy while already on ART [18], rapid scale‐up of optimized ART regimens, rates of viral suppression among pregnant and breastfeeding women on ART ranging from 30% to 98% in SSA [18] and 74.5% retention in care at 12 months [5]. While the benefit of infant prophylaxis in the context of suppressive maternal treatment is debatable, in real‐world settings, there are persistent issues that prevent all mothers living with HIV from achieving and maintaining viral suppression. The majority of new paediatric HIV infections continue to occur due to persistent gaps in the implementation of prevention of vertical transmission services. Infant infections now occur predominantly in the setting of incident maternal infections during pregnancy and breastfeeding, late maternal identification and ART initiation, and sub‐optimal ART adherence and retention. While the current approach to PNP cannot prevent transmissions related to maternal incident infections during breastfeeding, its role in reducing transmission for mothers who are newly identified, and not on suppressive ART has been documented in various settings [19].

Implementation challenges with enhanced and extended PNP implementation persist, these include individual, societal and structural barriers limiting effective implementation of risk stratification, multi‐drug regimens and prolonged prophylaxis, limited integration of PNP with postnatal services and poor follow‐up during breastfeeding.

While the possibility of not providing any PNP to the large number of infants born to mothers with consistent virologic suppression deserves evaluation, PNP remains an important tool in the toolbox to prevent vertical transmission as long as substantial gaps in maternal treatment implementation persist. In this context, it is essential to consider better strategies with tolerable, feasible, affordable agents that could facilitate the wider uptake of effective PNP.

New approaches for PNP may need to marry complementary strategies for PNP: ensuring a “safety net” with multi‐drug PNP at birth for those infants at high risk for perinatal transmission (that despite prophylaxis may acquire the infection and benefit from a triple regimen in alignment with treatment approaches); as well as a “safety belt” to preven postnatal transmissions by providing single‐drug prolonged prophylaxis through the breastfeeding period to protect the infant from potential episodes of maternal viremia. Given that prolonged PNP is most needed by infants born to mothers having difficulty maintaining adherence to daily ART, novel products and strategies with infrequent dosing will be critical to the success of a prolonged prophylaxis strategy.

### 2.4 Tailoring future PNP strategies to the programmatic context

**Figure 1:**
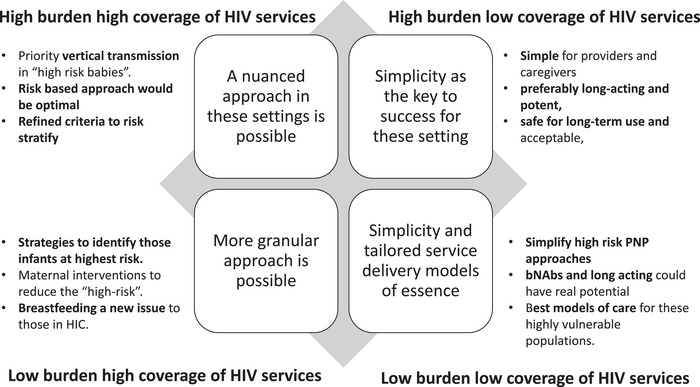
Postnatal prophylaxis based on setting and programme performance.

HIV programme maturity and epidemiological context may substantially influence our approach to optimizing PNP with attention to what is feasible, acceptable and to the desired impact. If we explore this diversity and attempt to tailor optimization of PNP to programmatic needs, we can conceptualize four scenarios: settings with high burden high coverage of HIV services, settings with high burden low coverage of HIV services, settings of low‐burden and high‐coverage HIV services and settings with low‐burden, low‐coverage of HIV services (Figure 1) bearing in mind that in some countries with considerable heterogeneity in the epidemic, policies on PNP might vary across sub‐national areas.

Participants convened during the first consultation of the WHO/IMPAACT workshop were asked to consider one of the four programmatic scenarios designed based on the burden and coverage of HIV services (particularly those to prevent vertical transmission). Through a number of predefined questions, each expert group engaged in a structured discussion, which was distilled down to the number of considerations for the design of improved PNP strategies. The outcomes of these discussions were then shared in plenary and refined with the input of the entire expert panel.

#### 2.4.1 PNP strategies in settings with high burden high coverage of HIV services

PNP needs to balance preventing HIV transmission with limiting ARV drug exposure to most infants not at risk for postnatal acquisition. In this context, the priority for PNP is to prevent residual vertical transmission by providing multi‐drug rescue prophylaxis to high‐risk infants who are born to women recently diagnosed with HIV or unsuppressed viral load. Extrapolating from clinical trial data in women not receiving ART [19], planned extended prophylaxis could be an option for infants defined at high risk for vertical transmission during breastfeeding because of specific concerns with maternal virological suppression. A risk‐based, for extended or enhanced prophylaxis, approach would be optimal to limit unnecessary drug exposure for infants who are exposed to HIV, uninfected and at low risk of acquiring HIV; however, there is a need for more refined and simplified criteria to stratify risk.

#### 2.4.2 PNP strategies in settings with high burden low coverage of HIV services

In settings with high HIV burden but low coverage of vertical transmission prevention services, low viral load and early infant diagnosis testing coverage, risk stratification is hindered by weak programme performance, hence, all infants exposed to HIV could be considered at high risk of HIV infection. Postnatal infant prophylaxis is critical in mitigating maternal testing‐to‐treatment gaps and high transmission risk. In this context, support should be given to strategies that attempt to simplify PNP implementation to better fit the health system challenges even if this may mean providing enhanced and or extended prophylaxis to infants who may not need it. For the greatest improvement in overall paediatric and maternal outcomes, health system deficiencies should be assessed and addressed.

#### 2.4.3 PNP strategies in settings with low‐burden, low‐coverage of HIV services

Often with concentrated epidemics (i.e. experienced within distinct sub‐populations): All babies in this category are high risk and eligible for enhanced PNP (with an “opt‐out” option). Simplification of approaches for high‐risk infants and tailored service delivery models will be of value for these settings. Newer technologies (e.g. broadly neutralizing antibodies and long‐acting ARVs) could be valuable in these settings. The research focus should be on the best models of care for marginalized populations—including people who use drugs, sex workers and undocumented and “hidden” populations—among whom the risk of vertical transmission occurs at many points along the continuum of care from identification through long‐term retention. Wider community engagement, partner and peer support are needed to promote the health of both mother and baby. The best opportunity of engaging this group into care is at the time of delivery, but current PNP regimens are not optimal for this purpose as they require good adherence. Long‐acting agents (either antiretrovirals or bNAbs) for PNP will be preferable in the long term. In the interim, simplified regimens should be explored. Solid formulations may be an option, including multiple drugs and integrase inhibitors. Health systems strengthening is recommended to identify at‐risk babies earlier.

#### 2.4.4 PNP strategies in settings with low‐burden, high‐coverage of HIV services

PNP for HIV in newborns continues to be based on postnatal oral zidovudine. Infants of mothers who do not have viral suppression in the weeks before delivery are considered high risk and—despite conclusive evidence of benefit—presumptive HIV therapy with three ARVs at therapeutic dosing is recommended. Infants of mothers who are on ART and have well‐controlled viral replication, especially those of mothers who are receiving ART at conception, are considered “low‐risk.” Currently, these “low‐risk” infants may receive anywhere from no PNP (Switzerland) to 4 weeks of zidovudine PNP (United States). Better refined risk stratification schemas may identify effective opportunities to further streamline PNP regimens and limit PNP beyond need based on risk. As the number of mothers with HIV in high‐resource settings who desire to breastfeed increases, it will be important to consider optimal PNP strategies for this scenario. A more granular approach is clearly possible but additional strategies to identify those infants at the highest risk might be needed as well as how minimal—if any—PNP might be required in those at low or very low risk.

## 3 CONCLUSIONS

There are several effective interventions that collectively reduce vertical transmission risk with postnatal ARV prophylaxis remaining an important component. Current WHO infant postnatal ARV prophylaxis approaches with risk‐based stratification rely on legacy ARVs that are already used for the treatment of HIV in infants and children and often (AZT) require twice daily administration (when AZT is utilized) and present operational challenges that prevent the achievement of the desired impact. Tailoring PNP strategies to programmatic context may improve access, adherence, retention in care and clinical outcomes of infants exposed to HIV. However, novel PNP approaches, using existing or future HIV agents, should ideally work well everywhere and possibly without risk stratification. These PNP strategies need to be simple, use drug regimens with low toxicity, and have the potential for shorter duration and limited dose changes. Existing service delivery platforms should be leveraged to promote integration with immunization and infant diagnosis, recognizing existing limitations within these systems and the need to identify effective approaches to address system failures. Newer ARV options and technologies prioritizing simplification of regimens, non‐toxic potent ARVs and convenient administration, including longer‐acting agents, should be considered and explored alongside their acceptability for caregivers and communities. Optimization of PNP remains a critical step not just to prevent vertical transmission but a key milestone on the path to reach elimination of vertical transmission and end the paediatric AIDS epidemic.

## COMPETING INTERESTS

The authors have no competing interests to declare.

## AUTHORS’ CONTRIBUTIONS

MP developed the outline. MP and IK developed the first draft of the manuscript. MP, EJA and TR finalized. All authors contributed to reviewing and editing the paper and approved the final draft.

## DISCLAIMER

The findings and conclusions in this report are those of the authors and do not necessarily represent the official position of WHUSAID, the US Government or the authors’ institutions.

## Footnotes

1 Botswana, Burundi, Cameroon, Cote d'Ivoire, DRC, Eswatini, Ethiopia, Ghana, Kenya, Lesotho, Mozambique, Namibia, Nigeria, South Africa, Tanzania, Uganda, Zambia and Zimbabwe.
